# Geographic profiling as a novel spatial tool for targeting infectious disease control

**DOI:** 10.1186/1476-072X-10-35

**Published:** 2011-05-18

**Authors:** Steven C Le Comber, D Kim Rossmo, Ali N Hassan, Douglas O Fuller, John C Beier

**Affiliations:** 1 Queen Mary University of London, School of Biological and Chemical Sciences, London E1 4NS, UK; 2 Center for Geospatial Intelligence and Investigation, Department of Criminal Justice, Texas State University, 601 University Drive, San Marcos, Texas, 78666, USA; 3 Institute of Environmental Studies & Research, Ain Shams University, Cairo, Egypt; 4 Department of Geography, University of Miami, Florida 33177, USA; 5 Department of Epidemiology and Public Health, Miller School of Medicine, and the Abess Center for Ecosystem Science and Policy, University of Miami, Florida 33136, USA

## Abstract

**Background:**

Geographic profiling is a statistical tool originally developed in criminology to prioritise large lists of suspects in cases of serial crime. Here, we use two data sets - one historical and one modern - to show how it can be used to locate the sources of infectious disease.

**Results:**

First, we re-analyse data from a classic epidemiological study, the 1854 London cholera outbreak. Using 321 disease sites as input, we evaluate the locations of 13 neighbourhood water pumps. The Broad Street pump - the outbreak's source- ranks first, situated in the top 0.2% of the geoprofile. We extend our study with an analysis of reported malaria cases in Cairo, Egypt, using 139 disease case locations to rank 59 mosquitogenic local water sources, seven of which tested positive for the vector *Anopheles sergentii*. Geographic profiling ranks six of these seven sites in positions 1-6, all in the top 2% of the geoprofile. In both analyses the method outperformed other measures of spatial central tendency.

**Conclusions:**

We suggest that geographic profiling could form a useful component of integrated control strategies relating to a wide variety of infectious diseases, since evidence-based targeting of interventions is more efficient, environmentally friendly and cost-effective than untargeted intervention.

## Background

Infectious diseases have, throughout human history, been a major cause of death. Just three such diseases - malaria, HIV/AIDS and tuberculosis - account for 5.6 million deaths a year, and neglected tropical diseases (NTDs) including leishmaniasis and trypanosomiasis are together responsible for 500,000 deaths annually [1]. Recent years have seen a resurgence in vector-borne diseases due to urbanization and development: Jones et al. [2] reported the emergence of 335 infectious diseases between 1940 and 2005.

Evidence-based targeting of interventions is a crucial component in the fight against infectious diseases because targeted interventions are more efficient and more cost-effective than untargeted interventions [3]; evidence-based interventions also reduce environmental pollution, minimize impacts on biodiversity and protect human health. For example, malaria parasite transmission is strongly dependent on the location of vector breeding sites that serve as focal points for the disease. Most transmission only occurs within certain distances from these sites; in Africa, these distances are typically between a few hundred meters and a kilometer, and rarely exceed 2-3 km [3]. Because of this clustering, untargeted intervention is highly inefficient. Accurate information on the distribution of malaria cases allows interventions to be focused at the points of transmission, greatly increasing the effectiveness of control measures [3]. Although source reduction of mosquito larval habitats can dramatically mitigate malaria transmission [4-7] the transient nature and diversity of potential vector breeding sites makes the identification and control of breeding sites difficult [3]. Consequently, the development of novel spatial tools that allow identification of infectious disease sources is of enormous interest [8].

Although spatial mapping of epidemiological data is well established (for example, see [9,10]), the emphasis is primarily on identifying spatio-temporal clusters of cases that suggest the possibility of outbreaks of disease [11-16], rather than on identifying sources of transmission in the early stages of outbreaks when such knowledge might be used to improve targeting of interventions, or depend on control data (e.g., [17,18]). Ostfeld et al. [19] highlight the complexity of vector-borne disease risk mapping by showing how the invasion of North America by West Nile Virus was not related to changes in the distribution or abundance of mosquitoes, but appears to follow changes in the distribution of WNV in vectors and avian reservoirs [20]. Consequently, Eisen and Eisen [8,21] identified a need for reliable new methods to determine probable pathogen exposure sites. Geographic profiling (GP), a statistical tool originally developed in criminology [22] and now beginning to be applied to biological and epidemiological data [23-26], may provide a means to directly identify sources of infection based on disease case locations.

In criminology, GP inputs the locations of connected crime sites, and uses these to make inferences about the most likely area of offender residence. The methods underlying GP depend on two concepts, (i) distance decay and (ii) the buffer zone [22]. Distance decay results from the fact that travel requires effort, time and/or money, and that most crimes thus tend to occur relatively close to the criminal's home; for example, 70% of arsons occur within two miles of a serial arsonist's home [27]. Similar constraints operate in infectious disease epidemiology where the probability of transmission declines with distance from an infected host [19]. The buffer zone, in criminology, is an area around the criminal's home in which offences are less likely, arising partly because of increases in detection risk related to reduced anonymity within the criminal's local neighborhood, and partly because the number of criminal opportunities increases with distance from home. The latter influence also occurs in biological systems: assuming suitable habitats are randomly dispersed throughout the surrounding area, then as the distance from the anchor point (usually a home or workplace) increases, the total number of suitable habitats increases. GP uses these opposing effects - the buffer zone and distance decay - to calculate the probability of offender residence for each location within the study area, producing an offender residence probability surface from the point pattern of the crime locations; this is referred to as a jeopardy surface. When the jeopardy surface is overlaid on a map of the search area, the result is a geoprofile. The higher a given surface point the greater the likelihood of offender residence for the underlying position. A geoprofile thus describes an optimal search process based on decreasing probability density, rather than highlighting a single area. Consequently, the performance of a geoprofile can be measured by the hit score percentage (HS%), the proportion of the area covering the crimes (or disease incidents) in which the offender's base (or disease source) is located; in criminology, this is usually the area bounding the crimes, plus a 'guard rail' or buffer of an additional 10%. The smaller the HS%, the more accurate the geoprofile; a hit score of 50% is what would be expected from a nonprioritized (i.e., random or uniform) search [22].

GP has been extremely successful in criminology, and it is now used routinely by law enforcement agencies around the world, including the Royal Canadian Mounted Police (RCMP), Scotland Yard and the Bureau of Alcohol, Tobacco, Firearms and Explosives (ATF). This success [28,29] led us to apply it to biological data [23-25]. Here, we extend this work to show how the technique may be applied to epidemiological data, using first John Snow's classic analysis of the 1854 London cholera outbreak [30], and second a series of malaria cases from Cairo, Egypt between 2001 and 2004. Specifically, we ask whether geographic profiling can use disease case locations to identify infection sources as a means of improving the targeting of interventions.

## Results

### London cholera study

**Figure 1:**
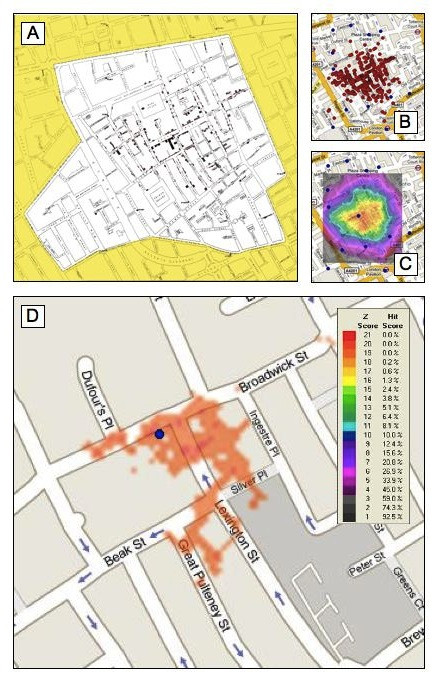
**Cholera and the Broad Street pump**. Cholera and the Broad Street Pump. John Snow's classic study of cholera cases in Soho in 1854 (A), and the same data plotted on a modern map of London, with red circles showing 321 individual addresses from Snow's original map and blue squares the locations of 13 neighbourhood water pumps (B). (C) The resulting geoprofile, omitting for clarity the locations of disease cases. (D) The top 1% of the geoprofile, clearly identifying the Broad Street pump.

A geographic profile prepared from 575 cholera deaths from 321 individual addresses from Snow's original data was used to evaluate 13 neighbourhood water pumps. The Broad Street water pump, the source of the cholera epidemic, ranked highest (HS% = 0.2%) (Figure 1). In this analysis, f and g were set to 1.2, the standard values in criminology. B is dynamic and is a function of the particular point pattern under analysis, and is set to half the mean nearest-neighbour distance in order to address scale issues; here, B = 53 feet.

### Cairo malaria study

**Figure 2:**
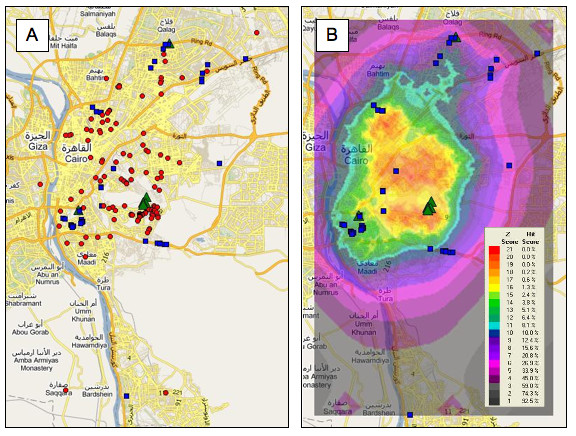
**Malaria cases and *Anopheles sergentii *breeding sites in Cairo**. Malaria cases and *Anopheles sergentii *breeding sites in Cairo. Map of Cairo showing locations of 139 recorded *Plasmodium vivax *malaria cases (red circles) and eight water sources that tested positive for the malaria vectors *An. sergentii *and/or *An. pharoensis *(green triangles); 51 other water sources that were negative for anopheline vector species are shown as blue squares. (B) the resulting geoprofile, omitting locations of malaria cases. The seven sites positive for *An. sergentii *ranked 1-6 and 22 out of 59 in our analysis.

We also analysed the occurrence of malaria in Cairo, Egypt, between 2001 and 2004, and used geographic profiling to rank 59 bodies of water harbouring at least one mosquito larva over the study period. Of these 59 sites, eight tested positive for one or both of the malaria vectors *Anopheles sergentii *and *An. pharoensis *(*Anopheles sergentii *is well established as the most dangerous malaria vector in Egypt [31]). Geographic profiling ranked six of these - all positive for *An. sergentii *- in positions 1-6, in the top 2% of the geoprofile. The other two positive sites were ranked 22 and 44; the last of these was positive for *An. pharoensis*, but not *An. sergentii *(this was the only positive site that did not contain *An. sergentii*). Overall the eight breeding sites in which the vector species were identified were ranked more highly than the other sites (mean ranks 10.9 and 33.0 respectively) and had lower hit scores (medians 0.85% and 35.88% respectively; Mann-Whitney U test: W8,51 = 87.5, P < 0.001) (Figure 2). Again, f and g were set to 1.2; here, B = 1,214 feet.

### Geographic profiling versus other methods of spatial central tendency

In both of our studies, geographic profiling proved highly efficient in identifying sources of infection using disease case locations when compared to other methods such as spatial mean, median and centre of minimum distance. In the London cholera analysis, the model's hit score was 0.2%, compared to 5.1%, 7.4% and 5.2% for the other three methods respectively. In the Cairo analysis, geographic profiling performed better than all three other methods for five out of seven sources, and was almost the best for one of the other two sources (4.46% versus 4.41%). Only for one source, which happened to lie close to the centre of the disease case locations, did the model not perform as well. Overall, the mean score for the seven sources was 2.77% for the geographic profiling model, versus 4.46%, 3.99% and 4.43% for the other methods respectively (Table 1).

**Table 1:** Geographic profiling and other measures of spatial central tendency

	Geographic profiling	Other methods of spatial central tendency		
**Site**	**Hit score**	**SSA (Spatial mean)**	**SSA (Spatial median)**	**SSA (Centre of minimum distance)**
**(a) London**				
**Broad Street pump**	0.20%	5.10%	7.40%	5.20%
**(b) Cairo**				
**Source 1**	2.80%	4.50%	4.00%	4.40%
**Source 2**	1.30%	5.87%	5.35%	5.85%
**Source 3**	2.18%	0.42%	0.11%	0.38%
**Source 4**	2.53%	5.17%	4.68%	5.14%
**Source 5**	3.06%	5.22%	4.72%	5.19%
**Source 6**	3.06%	5.16%	4.67%	5.13%

A comparison of the geographic profiling model's performance against measures of spatial central tendency for (a) the London cholera data and (b) the Cairo malaria data. For each source of infection (the Broad Street pump in the London analysis, and the seven water sources that tested positive for *An. sergentii *in the Cairo analysis), the table shows the model's hit score percentage in comparison to the spiral search area (SSA) percentage for three measures of spatial central tendency, with the most efficient search marked in red. The SSA is obtained by calculating the percentage of the total area under consideration that has to be searched before locating the correct source, moving out equally in all directions from, respectively, the spatial mean, spatial median and centre of minimum distance; it is thus equivalent to hit score percentage in geographic profiling (see *Methods*). In the London analysis, GP located the correct source after searching a smaller percentage of the surrounding area (0.2%, compared to 5.1%, 7.4% and 5.2% for the other three methods respectively). In the Cairo analysis, geographic profiling performed better than all three other methods for five out of seven sources, and was almost the best for one of the other two sources (4.46% versus 4.41%). Only for one source, which happened to lie close to the centre of the disease case locations, did the model not perform as well.

## Discussion

We suggest that geographic profiling could form a useful component of integrated control strategies relating to a wide variety of infectious diseases, by improving targeting of interventions. This is important because of the dramatic improvements in efficiency and cost-effectiveness of targeted interventions compared to untargeted interventions [3].

GP offers a number of advantages over other measures of spatial central tendency. Direct comparisons of the predictive capability of the Criminal Geographic Targeting (CGT) algorithm used in geoprofiling to the spatial mean show the former approach is both more accurate and more consistent, generating a search strategy approximately three times as efficient, and with a smaller standard deviation [32]. The CGT algorithm has also proved to be both robust and generalizable to a range of crimes in various environments. One important advantage of GP is that it explicitly allows for differences in landscape permeability, in the same way as cost surface analysis considers least-cost pathways. Also, because GP produces a probability score for each point in the study area, it describes an optimal search process as opposed to a single 'central point'. This is useful (i) when there are multiple anchor points (for example in the malaria analysis, where spatial mean, spatial median and centre of minimum distance performed well in identifying one source that occurred near to these points, but more poorly than geographic profiling for the others); and (ii), when differences in landscape permeability have skewed the location of the crime sites (for instance, when travel is more rapid along major roads). In biology, these are analogous to outbreaks with multiple sources, and variance in transmission probability in different habitats. A further advantage to the GP approach in both spatial criminology and spatial epidemiology is that the data are not constrained to administrative units such as census tracts, which often contain heterogeneous environmental conditions, but can utilise point observations. We know, for example, that offenders (or disease vectors) will seek nearby similar opportunities if their preferred criminal opportunity (or blood meal) is blocked or removed [33]; so a method like GP overcomes the limitations of spatially aggregated data by revealing continuous patterns and processes that might be masked when using administrative units as map inputs (this is a common problem in risk mapping that incorporates both socioeconomic and biophysical data). A final advantage to GP is the possibility of post-hoc analysis; i.e., a geoprofile could become a useful device to gauge the effectiveness of interventions after a specified time lag if the location of interventions is known and subsequent case locations are used to generate a new geoprofile after the application of an intervention, such as residual indoor spraying or use of insecticide-treated bed nets.

Buscema et al. recently applied the geographic profiling to the same cholera data set studied here [26]. However, the geographic profiling algorithm was not applied correctly, failing to locate the Broad Street pump; as we show here, correct application of the model does locate this pump. In fact, interpretation of the results in this study is complicated by the authors' use of error distance, rather than hit score percentage, to measure model performance. Error distance is a flawed metric, reducing all of the information in a geoprofile to a single point. In fact, one of the great strengths of geographic profiling is that it produces a surface describing a search strategy, rather than a point estimate: clearly, if the actual source occurs at (to take a trivial example) the second highest point on the geoprofile, the error distance between the peak of the geoprofile and the source is immaterial, since the search strategy described by the geoprofile will still be highly efficient - in this case, locating the source after searching just 0.02% of the study area (ie two points in the 100 × 100 matrix produced by Rigel). In addition, the 'hidden unit' point estimate the authors favour is poorly suited to cases where there are multiple sources. As our analysis of the Cairo data shows, geographic profiling deals well with such cases.

The results reported here are highly encouraging; however, this study represents only the first step in applying GP to epidemiological studies of infectious diseases. Clearly, epidemiology differs from criminology in a number of obvious ways - and presumably in some ways that are not yet obvious - and further studies will be required to examine how best to implement GP in this new field. As an example of such an adjustment, recent work using GP to study counterinsurgency has shown that that geoprofiles of IEDs (improvised explosive devices) are much more likely to identify the location of the bomb maker than the bomb placer [34,35]. Even beyond the differences between criminology and epidemiology, infectious diseases differ widely, and different diseases, or types of diseases, may require different approaches within the framework of GP. All this remains to be investigated.

One obvious difference between epidemiological data and criminal data is that in the former the original sources of infection can give rise to secondary sources of infection. Such spatio-temporally dynamic approaches are relatively common in epidemiology; for instance, research on foot and mouth disease (FMD) in the UK [19]. Similar approaches have been used in criminology to identify changes in temporal patterns of crimes. Clearly, it would be interesting to extend the approach described here to include longitudinal studies to establish whether case data can be used to identify secondary, as well as primary, sources of infection.

Rossmo [22] lists a number of operational procedures for the use of geographic profiling in a criminal investigation, including necessary assumptions and minimum data requirements. For example, he notes that different crime locations (e.g., place of victim encounter, murder scene, body dump site) have different theoretical meanings and have to be analyzed appropriately. Similarly in epidemiology, it would be important to distinguish between different location types (e.g., the capture site of a disease carrying insect, the home address of a sick individual, the location of an infected animal). Certainly if geographic profiling is to enjoy the same success in epidemiology that it has enjoyed in criminology, detailed examination of these issues in a range of different types of infectious disease is required.

Fortunately, the application of geographic profiling to the control of infectious disease need not start from scratch. There are a number of different geographic profiling software programs available, including Rigel [22,36], developed by Environmental Criminology Research Inc. (ECRI), CrimeStat [37], funded by the U.S. National Institute of Justice, and Dragnet [38], developed at The University of Liverpool. Rigel and Dragnet are commercial programs.

In addition to these general points, our analyses here, in combination with previous biological studies [23-25] and the extensive literature on geographic profiling in criminology, suggest a number of factors to consider if geographic profiling is to be applied to epidemiological data. Fortunately, most of the likely problems have analogues in criminology, and in many cases there are standard methods that minimise their effect. For example, edge effects can complicate the analysis. In criminology, this is usually compensated for by extending the minimum bounding rectangle encompassing all of the crime sites to include a 'guard rail' or cushion around the study area. Similarly, vectors such as mosquitoes may disperse more easily across some types of environments than others; however, such non-isotropic surfaces (or data collection) are common in criminology, where movement along major roads may be considerably more rapid than movement in other directions. Other difficulties - such as spatial or temporal non-independence of locations - are also well understood in criminology. In fact, epidemiology may in some instances (if not in others) present fewer difficulties than criminology; for example, while missing data are likely in both fields, epidemiologists do not have the problem of criminals actively attempting to mislead them. Another obvious difference is that in the analyses reported here, the geographic profiling was used to rank potential foci that had already been identified (water pumps and standing water). This is similar to the situation in criminology, where the method is primarily used to rank existing lists of suspects; however, the method can also be used to identify potential foci from the surface of the geoprofile, with no other information.

Our primary aim in this paper is to show how a technique that has been successful in criminology can be applied to epidemiology, but of course this cross-fertilisation can also work in the opposite direction. For example, while in criminology work has focussed on spatial central tendency, this is not the case in epidemiology. Epidemiological approaches can - very broadly - be divided into three main classes; (i) Dynamic, deterministic or stochastic models; (ii) Use of environmental correlates to map disease risk; (iii) Risk mapping using disease incidence. Dynamic, deterministic or stochastic models have been used extensively to analyse the factors that govern the spatial patterns, processes of diseases and to assess the effects of various interventions to stem the spread of infectious agents [39]. Traditional epidemiological approaches can be made spatially explicit by including network models, multigroup models, distance-transmission models and patch models [40]. Our GP study is most closely related to the distance-transmission modelling in that it employs buffers and a distance decay functions based on vector and case data where the spatial coordinates are known. Further, both types of disease transmission highlighted in our study can be related to environmental factors, which is a common way to evaluate disease risk [19]. The former relates to the presence of faulty urban infrastructure or poor sanitation, which is often difficult to spatially pinpoint a priori, whereas the latter relates to presence of infected vectors (anophelines) that are spatially heterogeneous and highly sensitive to microclimate and other abiotic factors such as breeding habitats (i.e., water bodies or containers). However, a major limitation to the use of environmental correlates is that direct causal relationships linking environmental conditions (e.g., poor infrastructure, substandard housing, poor sanitation, polluted water sources, climate, etc.) to disease incidence are often difficult to establish and may lead to spurious associations. Spatial criminology and infectious disease epidemiology share several characteristics: disease and crimes tend to be clustered near certain land uses or other environmental features, both criminals and disease agents (e.g., vectors) do not often travel far, and there is often a distance-decay effect between the source of the disease (or crime) and environmental features associated with either disease or crime [41].

For some diseases such as malaria and cholera, accurate spatial data exist on the location of cases, but spatial data are more difficult to obtain for vectors, their reservoirs, pollution sources or related environmental factors. Spatial data on disease incidence can be used to extrapolate the risk of disease exposure (or crime) from present distributions to areas outside the current, known distribution. Therefore, risk maps (or geoprofiles), can be seen as incorporating spatial variation of all risk factors including human-to-human exposure, contact with infected vectors, presence of disease reservoirs, etc. [19]. The GP approach within spatial epidemiology essentially falls under the broad rubric of risk mapping in so far as point observations are used to generate surfaces that indicate where vectors or pathogens are likely to be present. An advantage to using epidemiological data in mapping and modelling infectious disease is that maps represent actual risk [42], whereas maps based on environmental correlates are likely to represent potential risk. Of course, risk can be a poor predictor of incidence owing to use of interventions (e.g., bednets or water filtration in the case of malaria and cholera, respectively) or herd immunity (e.g., in the case of influenza and other vector-borne diseases such as dengue). Also, surveillance methods and discrepancies between exposure location and the location of the reported case may complicate further the spatial relationships between risk and incidence. Notwithstanding these limitations, the extension of the GP approach from criminology to spatial epidemiology appears quite powerful in the case where environmental data are scant and/or causal mechanisms are unknown. In this sense, the application of GP seems most appropriate in the context of new and emerging infectious diseases where causal agents or their environmental correlates (or determinants) are unknown, but may be ascertained by examining environmental data in the context of geoprofiles.

## Conclusions

In conclusion, we suggest geographic profiling has the potential to form a useful component of integrated control strategies for a wide variety of infectious diseases, including those with a mobile vector (as in our Cairo study) and those spread from a point source (as in the cholera study).

## Methods

### Model

For each point (i,j) in the study area, the score function p is calculated as follows:(1)

where(2)

and(3)

such that φ functions as a weighting factor that is set to 0 for disease case locations within the buffer zone, and 1 for locations outside the buffer zone. k is an empirically determined constant, B is the radius of the buffer zone, C is the number of disease cases, f and g are empirically determined exponents, (xi,yj) are the coordinates of point (i,j) and (xn,yn) are the coordinates of the nth location. Thus, pij describes the likelihood that the anchor point occurs at point (i,j), given the locations of the disease cases [22].

### London cholera data

Snow [30] collected address data on 575 cholera deaths (it would have been preferable to work with all known cases, not just mortalities). We digitized and geocoded the locations of 321 individual addresses from Snow's original map (as many of the deaths occurred in the same building (mean 1.79; range 1-18), addresses were used instead of cases to avoid the problem of spatial-temporal non-independence resulting from secondary contagion).

### Cairo malaria data

As part of another study, spatial data relating to 139 recorded *Plasmodium vivax *malaria cases were collected, and buffer zones of 2 km were created around the locations of these malaria cases and merged to form a polygon of 296.5 km^2^. All accessible aquatic habitats within this study area (surface/cryptic; temporary/semi-permanent/permanent) were located and characterised between April and September 2005. These included water tanks, water pools created through pipelines or drainage system breakage, seepage from slum housing, natural springs, pools and ditches filled with ground water. Water sources included in this analysis were identified as bodies of water harbouring at least one mosquito larva over the study period (n = 59). A total of 11 mosquito species were identified, including the malaria vectors *An. sergentii *and *An. pharoensis*, as well as other, non-vector, species.

### Geoprofiling

We analyzed the disease cases discussed in this article following the same standard procedures used in the preparation of a geographic profile for a crime series and using the software Rigel [22,36]. To compare the model's performance to a simple estimates of spatial central tendency, we compared the model's hit score percentage to the spiral search area (SSA) percentage, which is obtained by calculating the percentage of the total area under consideration that has to be searched, moving out equally in all directions from the spatial mean, spatial median and from the centre of minimum distance.

## Competing interests

DKR has a commercial relationship with ECRI, Inc, the manufacturers of Rigel. SLC, DOF, JCB and ANH declare that they have no competing interests.

## Authors' contributions

SLC, DOF, DKR and JCB conceived the study and wrote the paper. DKR constructed the geoprofiles. ANH provided data for the Cairo study. JCB was partially supported by the Abess Center for Ecosystem Science and Policy (CESP), University of Miami. ANH received funds from WHO/EMRO/TDR (project number SGS 110). The study was partially supported by NIH grant 5R01GM93345-2. All authors read and approved the final manuscript.
